# Hyperoxia does not directly affect vascular tone in isolated arteries from mice

**DOI:** 10.1371/journal.pone.0182637

**Published:** 2017-08-10

**Authors:** B. Smit, Y. M. Smulders, M. C. de Waard, H. M. Oudemans–van Straaten, A. R. J. Girbes, E. C. Eringa, A. M. E. Spoelstra - de Man

**Affiliations:** 1 Department of Intensive Care, VU University Medical Center, Amsterdam, the Netherlands; 2 Department of Internal Medicine, VU University Medical Center, Amsterdam, the Netherlands; 3 Department of Physiology, VU University Medical Center, Amsterdam, the Netherlands; Boston University, UNITED STATES

## Abstract

Hospitalized patients often receive oxygen supplementation, which can lead to a supraphysiological oxygen tension (hyperoxia). Hyperoxia can have hemodynamic effects, including an increase in systemic vascular resistance. This increase suggests hyperoxia-induced vasoconstriction, yet reported direct effects of hyperoxia on vessel tone have been inconsistent. Furthermore, hyperoxia-induced changes in vessel diameter have not been studied in mice, currently the most used mammal model of disease. In this study we set out to develop a pressure-myograph model using isolated vessels from mice for investigation of pathways involved in hyperoxic vasoconstriction. Isolated conduit and resistance arteries (femoral artery and gracilis arteriole, respectively) from C57BL/6 mice were exposed to normoxia (PO_2_ of 80 mmHg) and three levels of hyperoxia (PO_2_ of 215, 375 and 665 mmHg) in a no-flow pressure myograph setup. Under the different PO_2_ levels, dose-response agonist induced endothelium-dependent vasodilation (acetylcholine, arachidonic acid), endothelium-independent vasodilation (s-nitroprusside), as well as vasoconstriction (norepinephrine, prostaglandin F2α) were examined. The investigated arteries did not respond to oxygen by a change in vascular tone. In the dose-response studies, maximal responses and EC50 values to any of the aforementioned agonists were not affected by hyperoxia either. We conclude that arteries and arterioles from healthy mice are not intrinsically sensitive to hyperoxic conditions. The present ex-vivo model is therefore not suitable for further research into mechanisms of hyperoxic vasoconstriction.

## Introduction

Oxygen supplementation is frequently applied in modern day hospital care[1]. Superfluous administration of oxygen will lead to a supraphysiological oxygen tension in arterial blood (hyperoxia). Studies in healthy volunteers and hospitalized patients have shown that hyperoxia can influence the cardiovascular system by reducing cardiac output and increasing systemic vascular resistance[2–4]. Outside the haemoglobin compartment, dissolved oxygen hardly contributes to the oxygen delivering capacity of blood. The diminished flow due to reduced cardiac output and vasoconstriction may be disproportionate to the small increase in blood oxygen content, effectively impairing oxygen delivery to organs[5]. Additionally, increased oxidative stress may occur due to the abundance of molecular oxygen [6–8]. Both factors may be harmful for patients, which is corroborated by retrospective studies showing a correlation between high oxygen tensions and mortality in ICU patients [9–13]. Two small prospective ICU studies also showed increased mortality in patients with hyperoxia[14,15].

The increase in systemic vascular resistance is thought to reflect systemic vasoconstriction. Hyperoxic vasoconstriction has been investigated in preclinical studies using a variety of animal models (e.g. dogs[16–19], pigs[20–27], cats[20,28,29], rats[30–39], rabbits[40], sheep[41], hamsters[42–51] and mice[52–54]) using intravital microscopy and isolated vessel myography. However, despite decades of research, the mechanisms of hyperoxic vasoconstriction remain elusive [55]. Although the majority of studies suggest constriction, some show no direct effect of hyperoxia on vascular tone[27,29,34,38], while others even show dilatation[16,18,23,41].

A mouse model for hyperoxic vasoconstriction, utilizing vessel myography, would be highly valuable due to the possibility of using genetically modified animals. Vessel myography is an excellent tool to explore vascular pathways because systemic variables that influence blood vessel tone (like blood pressure, sympathetic activation, blood components) can be excluded. However, no isolated vessels from mice have been used in either a wire or pressure myography setup to study the effects of hyperoxia.

In this study, we aimed to design a pressure myography model for direct effects of hyperoxia on artery diameter using isolated arteries from healthy mice, to enable use of gene-knockout mouse strains, for future investigation of the pathway(s) involved in hyperoxic vasoconstriction.

## Methods

### Animals

In all experiments we used 10–12 week old male C57BL/6 mice, maintained on standard chow and water, ad libitum. Animals were housed in the Amsterdam Animal Research Centre until used for the experiments described below. All protocols were reviewed and approved by the Animal Experiments Committee of the VU University, Amsterdam.

### Isolation of vessels

Mice were killed by cervical dislocation. The femoral artery and its first order arteriole feeding the gracilis muscle (gracilis arteriole) were isolated from both hind legs immediately. During the procedure, the area was kept cold and moist by intermittent dripping with ice-cold MOPS buffered physiological salt solution (PSS) containing (in mM): 145 NaCl; 4.5 KCl; 2.5 CaCl_2_; 1.2 MgSO_4_; 1.2 H_2_NaPO_4_; 0.025 EDTA; sodium pyruvate; 11 glucose; MOPS. All isolated vessels were stored on ice until cannulation.

### Cannulation of vessels

Vessels were transferred to a pressure myography adapted for direct administration of gases to blood vessels, filled with 10 ml of Krebs-Henseleit buffer (KHB) containing (in mM): 119.0 NaCl; 4.7 KCl; 2.5 CaCl_2_; 1.2 MgSO_4_; 20.0 NaHCO_3_; 1.2 KH_2_PO_4_; 0.025 EDTA; 11 glucose. The buffer was warmed to 37°C and gassed with a mixture of 10% O_2_, 5% CO_2_, balance N_2_ to obtain a pH of 7.4 and PO_2_ of ~80 mmHg. Oxygen tension of the buffer surrounding the vessel was measured with a Fibox 4 oxygen meter (PreSens, Regensburg, Germany). Vessels were mounted on glass cannulas on either side, secured with a single suture and stretched slightly in the longitudinal direction. Intravascular pressure was raised to 80 mmHg and the vessel segment was studied without intraluminal flow.

### Experimental protocol

Vessels were allowed to rest for 20–30 minutes before starting the experiments. After this period, diameters were stable. Vessels that did not develop spontaneous myogenic tone were constricted to 40–50% of their initial diameter with norepinephrine 10^−6.5^ M (Centrafarm BV, Etten-Leur, the Netherlands). Vessels exhibiting spasms were discarded. When a stable constriction was obtained, endothelial integrity was tested using 10^−7^ M Acetylcholine. Vessels had to respond with a stable dilation of at least 10% and were discarded otherwise.

The buffer in the vessel chamber was then replaced with fresh KHB and gassed with either 10, 30, 50% or 90% O_2_ / 5% CO_2_ / balance N_2_ to reach a PO_2_ of approximately 80, 220, 350 or 660 mmHg. After endothelial integrity was verified, 10, 30 or 50% oxygen was applied randomly and each acted vessel as its own control. Experiments with 90% O_2_ were performed in a later stage. For clarity, the different groups in this manuscript will be named according to the percentage of oxygen used.

Semi-log cumulative dose-response curves were established for Acetylcholine (10^−9^–10^−5.5^M) to test the eNOS pathway, S-nitroprusside (10^−9^–10^−5.5^M) for vascular smooth muscle sensitivity to Nitric Oxide and Arachidonic Acid (10^−9^–10^−5^) to study cyclooxygenase dependent dilation. Norepinephrine (10^−9^–10^−5.5^M) and Prostaglandin F2α (10^−9^–10^-5^M) were applied to test sensitivity to naturally circulating constrictors. Maximal diameters were obtained with application of 10^-4^M Papaverine. All chemicals, except for norepinephrine (Centrafarm BV, Etten-Leur, the Netherlands) were purchased from Sigma-Aldrich (Zwijndrecht, the Netherlands).

### Statistical analysis

At each dose, dilation or constriction was calculated as a percentage of the maximal response margin [56,57]. If multiple vessels were used from one animal for the same agonist and oxygen tension, the results were averaged. Therefore, numbers reported refer to animals used, rather than individual vessels. Using GraphPad prism, a non-linear four parameter fit of the data was performed for calculation of the EC_50_ variable. Maximal responses and EC_50_ values where compared with one-way ANOVA and Dunnett’s post-hoc correction for multiple comparisons where appropriate. Values are presented as mean (standard deviation) unless otherwise specified.

## Results

A total of 48 mice were used to obtain the results described below. On average, the mice weighed 25 gr (3).

The oxygen tensions measured during the experiments when gassing with either 10, 30, 50 or 90% O_2_ were 78 mmHg (4), 215 (6), 375(26) and 667(5), respectively. The femoral arteries used in the experiments had a maximal diameter of 293 μm (30). Gracilis arterioles were 129 μm (20) wide.

Femoral arteries did not develop spontaneous tone. For dilation studies (Acetylcholine, S-Nitroprusside and Arachidonic Acid), the femoral arteries were therefore constricted to 46% (12) with norepinephrine. The average tone for gracilis arteries was 42% (15).

We found no effect of oxygen on the baseline vessel diameter. For the gracilis arterioles, the degree of tone was therefore similar between the different oxygen tensions; 40% (16), 40 (14), 43 (15) and 40 (18) at a PO_2_ of 80, 215, 375 and 665 mmHg respectively (P = 0.72).

**Fig 1:**
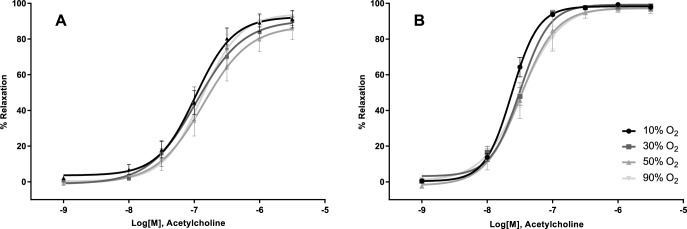
Effect of oxygen on acetylcholine induced dilation. The endothelium dependent vasodilation of the Femoralis (A) or Gracilis (B) to acetylcholine was not influenced by hyperoxic oxygen tensions. Acetylcholine activates eNOS to produce Nitric Oxide.

**Fig 2:**
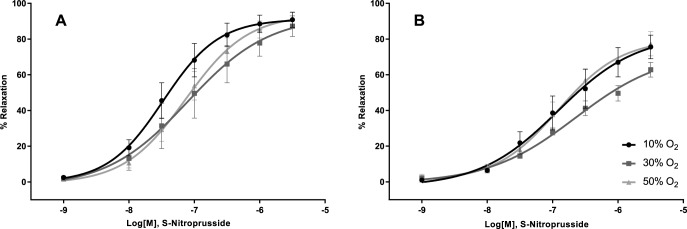
Effect of oxygen on S-nitroprusside induced dilation. Endothelium independent dilation was not influenced by oxygen tension in either the Femoralis (A) or Gracilis (B).

**Fig 3:**
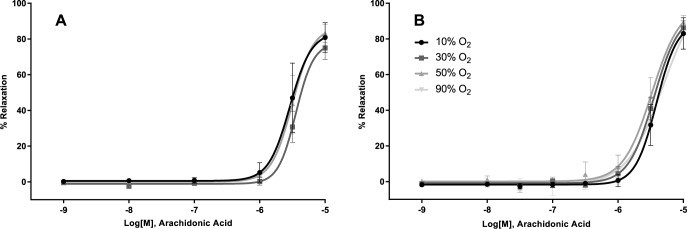
Effect of oxygen on arachidonic acid induced dilation. Arachidonic acid produced similar responses under the different oxygen tensions used in both the Femoralis (A) and Gracilis (B). Arachidonic acid is metabolized in the endothelium to dilating and constricting prostaglandins through cyclo-oxygenase.

The dilation of the femoral artery or gracilis arteriole induced by application of acetylcholine, which activates eNOS to produce NO, was not affected by oxygen tension (Fig 1A; Femoralis, 1B; Gracilis). The curve statistics are summarized in Table 1. The sensitivity of the vascular smooth muscle of either vessel to the NO donor SNP was not altered by hyperoxia (Fig 2A and 2B, Table 1). Similarly, endothelium dependent dilation with arachidonic acid did not differ between oxygen tensions (Table 1 and Fig 3).

**Table 1:** Vasodilation statistics.

		Maximal response					logEC_50_				
		(% dilation)					(10^x^ M)				
	N	10%	30%	50%	90%	P	10%	30%	50%	90%	P
*Acetylcholine*											
Femoralis	7,7,6,5	91 (13)	90 (6)	86 (15)	93 (6)	0.74	-7.0 (.18)	-6.9 (.16)	-6.9 (.27)	-6.9 (.11)	0.70
Gracilis	4,6,5,5	98 (3)	98 (2)	97 (5)	97 (2)	0.86	-7.6 (.05)	-7.5 (.07)	-7.5 (.17)	-7.5 (.05)	0.21
*SNP*											
Femoralis	5,5,5	91 (10)	87 (13)	92 (8)	-	0.77	-5.5 (.32)	-5.4 (.68)	-5.5 (.30)	-	0.77
Gracilis	5,5,6	76 (14)	68 (17)	73 (14)	-	0.68	-5.4 (.62)	-5.5 (.48)	-5.5 (.37)	-	0.88
*Arachidonic Acid*											
Femoralis	6,5,6	81 (19)	75 (14)	83 (11)	-	0.68	-7.5 (.20)	-7.1 (.23)	-7.1 (.17)	-	0.17
Gracilis	5,5,5,5	83 (20)	86 (9)	89 (10)	82(15)	0.81	-6.9 (.31)	-6.6 (.13)	-6.9 (.22)	-5.3 (.54)	0.84

Maximual values are the responses measured at the highest agonist concentration. LogEC50 values are derived from a non-linear four parameter fit. n denotes the number of animals per group. Values are reported as mean(SD).

**Fig 4:**
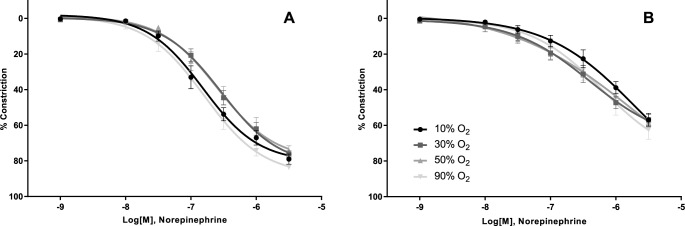
Effect of oxygen on norepinephrine induced constriction. Constriction through interaction with α and β adrenergic receptors was not modulated by hyperoxia in the Femoralis (A) or Gracilis (B).

**Fig 5:**
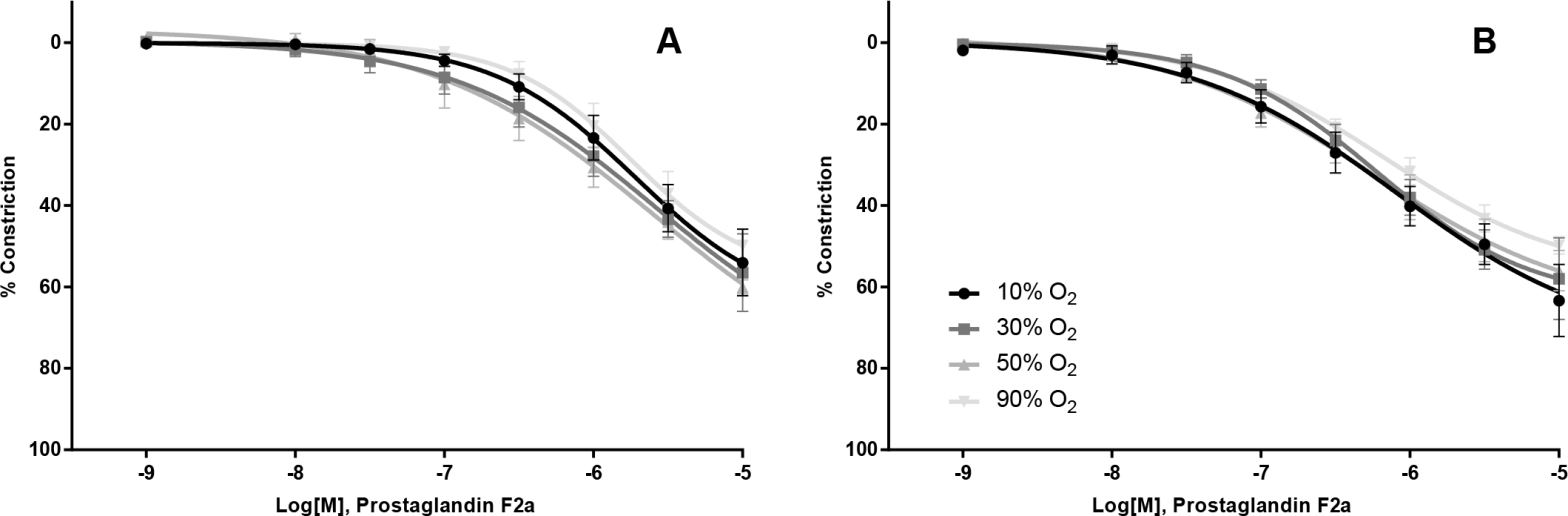
Effect of oxygen on Prostaglandin F2α induced constriction. The constrictor PGF2α caused similar levels of constriction under hyperoxic conditions in both the Femoralis (A) and Gracilis (B).

Constriction of vessels with increasing doses of norepinephrine (Table 2 and Fig 4) or Prostaglandin F2alpha (Table 2 and Fig 5) gave identical results under different PO_2_ levels.

**Table 2:** Vasoconstriction statistics.

			Maximal response					logEC_50_				
			(% constriction)					(10^x^ M)				
		n	10%	30%	50%	90%	P	10%	30%	50%	90%	P
*Norepinephrin*												
	Femoralis	5,6,5,5	79 (6)	76 (4)	74 (6)	83 (3)	0.35*	-6.8 (.20)	-6.5 (.21)	-6.6 (.25)	-6.8 (.19)	0.11
	Gracilis	5,5,5,5	57 (8)	57 (7)	58 (6)	62 (12)	0.69	-5.7 (1.6)	-6.4 (.55)	-5.7 (2.5)	-6.2 (.74)	0.86
*Prostaglandin F2α*												
	Femoralis	6,6,5,5	54 (14)	57 (13)	60 (3)	50 (9)	0.58	-5.7 (.68)	-5.6 (1.6)	-5.6 (1.5)	-5.7 (.40)	0.99
	Gracilis	6,6,5,5	63 (15)	58 (17)	56 (10)	50 (5)	0.46	-6.1 (.93)	-6.2 (.45)	-6.2 (.67)	-6.2 (.33)	0.98

Maximal values are the responses measured at the highest agonist concentration. LogEC50 values are derived from a non-linear four parameter fit. n denotes the number of animals per group. Values are reported as mean(SD).

*P value after Dunnet's correction for multiple testing (base P value 0.04).

## Discussion

In the present study, we have found that hyperoxia does not directly impair endothelium dependent and -independent vasodilation or vasoconstriction in isolated femoral arteries and gracilis arterioles from healthy C57BL/6 mice.

We found that different concentrations of oxygen did not change basal tone, endothelial or smooth muscle function in any of the arteries and arterioles studied. An inconsistent response of vessels to oxygen has been described previously in different setups and species, but no other data exists of isolated vessels from mice. In rats, arterioles from the cremaster muscle were found to be sensitive to oxygen in one study[30], but insensitive in another two[58,59], despite using the same species and a practical identical methodology. In vivo, increasing oxygen within the cremaster bed of mice induced vasoconstriction in response to elevated oxygen tensions in 60% (99 of 165 mice)[53]. Similarly, an elegant ex-vivo study on hamster cheek pouch arterioles showed that from the 28 arteries studied, only nine constricted in response to oxygen supplementation (32%)[42]. The authors could not find a specific factor that was responsible for the loss of oxygen reactivity in the remaining vessels. In particular, they ruled out damage to vessels as a cause due to similar myogenic tone and norepinephrine sensitivity between responding and non-responding vessels. All arteries and arterioles used in our study exhibited normal endothelium and smooth muscle function and therefore vessel damage is highly unlikely to be a factor explaining our results. Thus, while in the aforementioned experiments a proportion of arterioles responded to hyperoxia, none of the arteries/arterioles in our study did.

The lack of effect on vascular tone in our study strongly suggests that mouse arteries and arterioles are intrinsically insensitive to oxygen. Several explanations for this insensitivity can be hypothesized. For instance, mice could be insensitive to hyperoxia in general. This is likely not the case however, because in vivo, the cremaster muscle of mice has been suffused with hyperoxic buffer to induce vasoconstriction[52–54,60], suggesting that vessels from mice are capable of constricting to elevated oxygen tensions when the surrounding tissue is present.

The absence of cross-talk between tissue and the vessel wall is an obvious and intended factor in isolated vessel studies. Suggested extravascular cells that may influence vascular tone include mast cells, erythrocytes, striated muscle and perivascular adipose tissue (PVAT). Mast cells were found to adhere to the external side of the vessel wall of cheek pouch arterioles where they may produce constricting leukotrienes [55]. During the isolation of vessels, some or all mast cells may be removed or damaged, which could be an explanation for why in one study, only a portion of the studied vessels reacted to oxygen [42]. Perfusion of vessels from the brain with erythrocytes restored reactivity to oxygen [61], but they were found to be unimportant in cheek pouch [43] and cremaster preparations [53]. The Cytochrome P-450 ω-hydroxylase enzyme, which produces the vasoconstrictor 20-HETE under elevated oxygen tensions, is highly expressed in the rat cremaster muscle and the inhibition of the enzyme markedly reduced arteriolar sensitivity to oxygen [39]. Perivascular adipose tissue is a well-recognized modifier of vascular tone, releasing a plethora of vasoactive substances under healthy and inflammatory circumstances [62] and was found to modulate the sensitivity of isolated murine mesenteric arteries to hypoxia [63].

Vessel-order could be an important factor, because the vessels studied in vivo were considerably smaller than the smallest arterioles in our setup (~20 μm vs ~140 μm, respectively). Hence, it is possible that oxygen-dependent changes in vascular tone primarily occurs in higher-order arterioles. However, equally sized isolated vessels from rat have been reported to constrict to elevated oxygen tensions[30,32]. Also, ex-vivo, hyperoxic constriction was shown in large systemic arteries like the femoral artery[16], carotid artery[17], and abdominal[20,31] and thoracic aorta[20,31]. Hyperoxic vasoconstriction has also been shown in a plethora of resistance arteries originating from the gracilis[32], cremaster[30] and coronaries[19–22,24–27,64], although none of these were of mouse origin.

Another in vivo factor absent in our setup is intraluminal flow. Although flow induced shear stress is an important activator of the endothelium that causes a rise of intracellular calcium and a subsequent change in basal activity of phospholipase A2, cyclooxygenase and nitric oxide synthase [65], it is important to emphasize that hyperoxic vasoconstriction has been shown extensively in wire-myograph setups in which intraluminal flow is absent [17,20–22,25,26,31,64]. Also, out of the three pressure myography studies that used flow to investigate oxygen sensitivity, two did not add anything to the perfusate that increased the viscosity of the buffer (e.g. albumin or dextran), making actual shear stresses very low[30,32]. In our experiments, the most prominently involved pathways in shear induced dilation were tested with the addition of acetylcholine (NOS) and arachidonic acid (COX, PLA2). Taken together, it is unlikely that the absence of flow is a crucial factor in our experiments. Taken together, we are confident to state that femoral arteries and gracilis arterioles of mice are intrinsically not sensitive to oxygen. However, we want to express caution to extrapolate these results to other species and other vessels, considering that there appears to exist great heterogeneity in responsiveness to oxygen between species and vascular beds [55]. This also means that there is a possibility that currently established animal models for hyperoxia do not necessarily represent human physiology. Using human vessels is therefore perhaps a better option for future studies into hyperoxic vasoconstriction, which to our knowledge has been done only once [64].

A limitation to be considered is that we used only 4–7 animals per oxygen condition. However, we reduced measurement variation by using multiple vessels per mouse and averaging the responses. Since the overall variation was small, we do not think that the use of more animals would give different results.

Finally, there is a possibility of publication bias. From the dozens of papers on hyperoxic vasoconstriction, only two reported on the considerable oxygen-related difficulties experienced throughout the study [42,53]. Given the attention for cardiovascular effects of oxygen and the common use of resistance vessels from mice in myography experiments, it is unlikely that this was the first attempt to study hyperoxia in murine vessels. We strongly feel that it is important to report on models that give different results than expected. By publishing these data, future researchers are spared from fruitlessly spending effort and resources on this model for hyperoxia induced vasoconstriction.

## Conclusion

We conclude that arteries and arterioles from healthy mice are not intrinsically sensitive to hyperoxic conditions. The present ex-vivo model is therefore not suitable for further research into mechanisms of hyperoxic vasoconstriction.
